# BREEZE—Boundary Red Emission Zone Estimation Using Unmanned Aerial Vehicles

**DOI:** 10.3390/s22145460

**Published:** 2022-07-21

**Authors:** Oren Elmakis, Tom Shaked, Barak Fishbain, Amir Degani

**Affiliations:** 1 Technion Autonomous Systems Program, Technion—Israel Institute of Technology, Haifa 3200003, Israel; oren.elmakis@campus.technion.ac.il (O.E.); shakedtom@campus.technion.ac.il (T.S.); 2 Department of Environmental, Water and Agricultural Engineering, Faculty of Civil & Environmental Engineering, Technion—Israel Institute of Technology, Haifa 3200003, Israel; fishbain@technion.ac.il

**Keywords:** unmanned aerial vehicles, air pollution, gas mapping, catastrophic event, chemical leakage

## Abstract

Catastrophic gas leak events require human First Responder Teams (FRTs) to map hazardous areas (red zones). The initial task of FRT in such events is to assess the risk according to the pollution level and to quickly evacuate civilians to prevent casualties. These teams risk their lives by manually mapping the gas dispersion. This process is currently performed using hand-held gas detectors and requires dense and exhaustive monitoring to achieve reliable maps. However, the conventional mapping process is impaired due to limited human mobility and monitoring capacities. In this context, this paper presents a method for gas sensing using unmanned aerial vehicles. The research focuses on developing a custom path planner—Boundary Red Emission Zone Estimation (BREEZE). BREEZE is an estimation approach that allows efficient red zone delineation by following its boundary. The presented approach improves the gas dispersion mapping process by performing adaptive path planning, monitoring gas dispersion in real time, and analyzing the measurements online. This approach was examined by simulating a cluttered urban site in different environmental conditions. The simulation results show the ability to autonomously perform red zone estimation faster than methods that rely on predetermined paths and with a precision higher than ninety percent.

## 1. Introduction

Leakages in gas production or storage facilities are catastrophic events that can cause severe damage and human casualties by releasing toxic or flammable gases into the atmosphere. In the last decade, major gas leakage events have led to fatal results in Kaohsiung, Taiwan [1]; Chittagong, Bangladesh; and, most recently, in Visakhapatnam, India [2]. These events occurred suddenly due to insufficient maintenance, improper storage, or operation errors.

Gas leaks are not rare, as the prevalence of such events in the United States is ~630,000 leaks per year [3]. Each of these events requires a First Responder Team (FRT) to perform an immediate risk assessment. When the risk assessment indicates a threat to public safety, the FRT needs to estimate the area containing dangerous gas density (defined here as a ‘red zone estimation’) to guide the evacuation process. In this context, when attempting to prevent human casualties by evacuation, time is of the essence.

Currently, the FRT begins the red zone estimation process by performing predetermined path walkover surveys with hand-held gas detectors and by recording measurement data [4]. However, this process is prolonged by the limited capacity of human teams to perform real-time data analysis.

The urgency in such cases is currently met by compromising the Gas Dispersion Map (GDM) resolution, which runs the risk of producing biased or distorted information. This risk can be mitigated by performing smart mobile sensor placements to improve the measurements and to provide effective information for such tasks [5]. While this strategy can potentially reduce the necessary measurements required to produce an accurate GDM, it relies on a manual placement process, which poses a risk to the FRT.

Using robots that are capable of cooperating with the FRT on-site provides a solution to both problems stated above: robots equipped with onboard chemical sensing instruments can monitor gases in the search space and autonomously perform complex tasks without human intervention. The contribution of robot–FRT cooperation is in creating new possibilities for monitoring large-scale danger zones while improving the safety of humans in the process. Specifically, using gas-sensing robots to perform red zone estimation reduces the exposure of the FRT to dangerous gasses and their consequential effects.

Unmanned Aerial Vehicles (UAVs) are cost-effective and easy to deploy, making them a common robotic platform for gas-sensing operations. These platforms have three-dimensional maneuverability, allowing them to monitor areas with multiple obstacles while performing continuous gas sensing. While extensive work has been carried out in relation to algorithms for source localization and gas measurement analysis [6], there is a lack of methods for the efficient path planning of mobile sensors for mapping. In this context, the novelty of the presented paper is expressed by three elemental capacities: (1) developing an adaptive path-planning method for fast red zone estimation, (2) classifying the red zone and safe zone areas, and (3) providing an extensive simulative framework for examining gas-monitoring scenarios.

This paper presents a new adaptive path-planning approach for the effective mapping of red zones, termed here as **B**oundary **Re**d **E**mission **Z**one **E**stimation (BREEZE). This approach employs UAVs for gas sensing in urgent, catastrophic scenarios that require fast estimation of red zones during gas leakage events. Instead of mapping entire areas, the BREEZE approach classifies the map into safe-zone and red-zone regions. This approach saves significant time and energy by avoiding exhausting scans of the entire task zone. Specific focus is placed on overcoming the inconsistency of pollution density in building proximity as examined in the simulative framework. These capacities support the fast evacuation of civilians in emergency events in and around residential areas.

## 2. Related Work

Recent advancements in autonomous systems enable the use of robotic platforms for complex tasks traditionally carried out by humans. Unlike humans, robots are designed to perform repetitive tasks and online computations without suffering from fatigue. Furthermore, robots are less vulnerable to harmful substances and harsh environments than humans [7]. These capabilities enable them to analyze gas measurements online while employing adaptive path-planning strategies [8].

Unmanned Ground Vehicles (UGVs) can carry heavy payloads and operate for an extended period of time [9]. However, path planning for UGVs over rough terrain is a challenging problem that restricts these platforms to structured or well-known areas. In contrast, the 3D spatial maneuverability of UAVs allows them to freely operate over challenging terrains and in cluttered spaces while monitoring large areas [6]. This capacity makes them ideal for monitoring gas emissions both indoors [10] and outdoors [11].

The UAVs’ gas sensory capabilities are key to performing efficient and accurate monitoring. In this context, gas sensors are divided in their capacity for long-range and in-place sensing. Although long-range sensors provide a substantial advantage in their ability to sense the entire surrounding area [12], they are relatively large and have a high energy consumption, which substantially shortens the UAVs’ operation time. In contrast, in-place sensors are smaller and lighter [13], allowing them to minimize the onboard payload and therefore increase the UAVs’ operation time.

The fundamental problem in monitoring gases is finding an efficient path-planning strategy that overcomes the UAVs’ energy and payload limitations. The search for efficient methods is shown in path-planning strategies for gas source localization [14,15,16], which attempts to estimate the minimal path to a given emission source. However, this problem is exacerbated when the UAV needs to collect samples to create a precise GDM. A common approach for estimating the GDM suggests representing it as a discrete map, where each cell is assumed to contain a constant gas concentration [17]. This representation allows the implementation of probabilistic approaches that use sparse measurements to estimate the GDM [18]. In this context, the Gaussian kernel approach uses the Gaussian function as a weighting function [17] to efficiently estimate the map by assuming a regional connection between cells. Further efforts are then made to extend this method to overcome gas fluctuations over time [19] as well as to incorporate wind information [20]. 

Along with these representation approaches, which suggest ways to analyze gas measurements, an efficient path planner for gas sampling is required. While most studies use pre-planned trajectories [11], the path-planning objective is to find a sampling path that provides the best GDM estimation. One such approach suggests a reward function based on information theory quantities [21]. An alternative method for path planning suggests modifying an artificial potential field (APF) for navigating between sampling locations [22].

While the above path-planning approaches suggest mapping policies for estimating gas distribution in obstacle-free environments, there is a need for path planning in cluttered areas—in which the risk for humans is greater. Attempting to overcome this problem, the Gaussian Markov Random Field approach maps the task zone as a factor graph, connecting the safe path edges to produce a path that is free from obstacles [23].

As seen here, gas-monitoring tasks present three main challenges: (1) mapping gas distribution in real time, (2) finding efficient UAV paths while overcoming its limited time and payload capacity, and (3) monitoring gas in challenging environments such as cluttered urban areas. As shown in the related work, most studies employ pre-planned trajectories, which result in inefficient and prolonged monitoring processes. Moreover, existing research mainly focuses on obstacle-free areas. These areas do not represent common challenges typical to cluttered urban environments, such as gas distribution turbulency and having to navigate close to built elements.

The aforementioned studies attempt to solve the entire GDM problem, while the presented research suggests BREEZE—A novel approach to solving the red zone estimation problem by classifying the task zone into safe and dangerous areas. This action significantly improves task performance, specifically in cluttered environments.

## 3. Methodology

The following section provides the building blocks of this study, starting with a comprehensive explanation of the general system overview and then presenting the problem formulation with the metrics used to evaluate BREEZE performance.

### 3.1. System Overview

To examine the presented approach, the research includes developing a software simulation system. The proposed system aims to examine gas-monitoring strategies in a realistic simulation environment. To allow a comprehensive examination of the monitoring strategies, the framework employs (1) a Computational Fluid Dynamics (CFD) engine, (2) a rigid body dynamics simulator, and (3) dedicated software for system communication.

**Figure 1:**
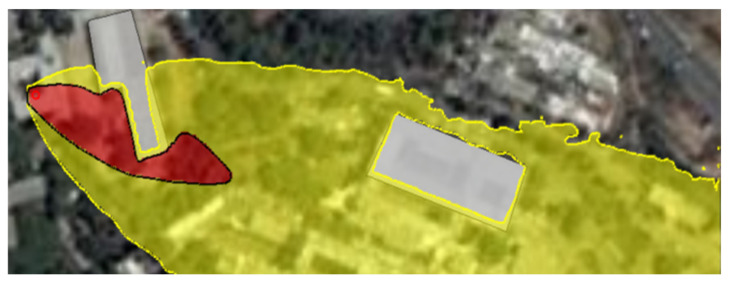
The Graz Lagrangian Model (GRAL) presents a ground truth simulation of gas dispersion with buildings. In red and yellow are the low-density and high-density gas areas.

**Figure 2:**
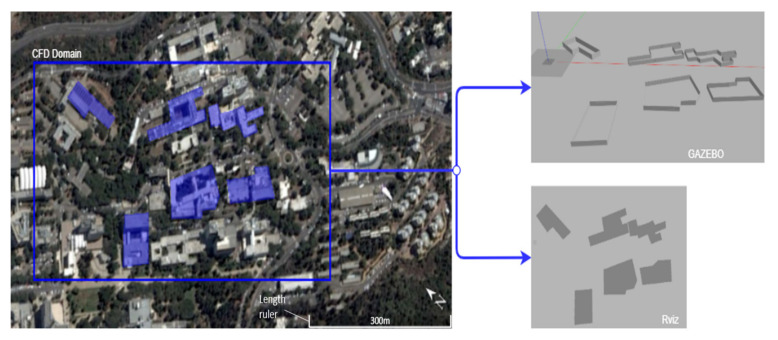
The six-building environment simulated in GRAL, Gazebo, and Rviz.

The Graz Lagrangian Model (GRAL) is a CFD engine that enables the generation of gas dispersion maps that include various obstacles such as buildings and vegetation in a simulated environment. Figure 1 presents a simulated environment generated using the GRAL CFD engine. The red and yellow areas in the figure delineate the high and low gas densities in the site, respectively. The site is located in the Technion—Israel Institute of Technology, and the buildings’ accurate coordinates were imported from Google Earth [24], as shown in Figure 2.

The rigid body dynamic simulator used here is Gazebo [25], an open-source simulator commonly used for robotic applications. The Gazebo UAV model is based on the RotorS package, which provides 3D models for UAV simulation, including odometry sensors and low-level control [26]. The use of Gazebo enables the system to examine the path feasibility—defined here as the capacity of the UAV to perform the path while considering the collision constraints on-site. 

Lastly, the communication between the system’s components is performed using the Robot Operating System (ROS)—A collection of frameworks for robot software development [27]. ROS enables the establishment of server–client communication and publisher–subscriber connections between the system’s components. ROS also includes the Rviz visualization library, which is used to monitor the progress of the UAV throughout the task.

### 3.2. Dispersion Simulations

The dispersion scenarios in this study are generated using GRAL (Figure 2). The GRAL gas dispersion simulation consists of a single pollution source with a gas emission velocity of 10 m/s, a temperature of 60 °C, and a diameter of 1 m. The simulated urban environment consists of a six-building site used to examine three wind conditions, each posing specific challenges to the red zone estimation process.

### 3.3. Sensor Simulation

The gas sensor component is located onboard the UAV, enabling online sampling during the task in each of the UAV’s positions along the path. The algorithm’s robustness is examined by tuning the sensor’s sampling rate and noise. The noise is embedded in the model as Additive White Gaussian Noise (AWGN):

(1) $$\;P\left(X^{i}\right)=\hat{P}\left(X^{i}\right)+\nu$$

where $X^{i}$ is the position of the UAV, and ν is the sensor’s noise.

### 3.4. Problem Formulation

The problem space is defined as a 2D cartesian grid M. The grid includes traversable cells (open space) and non-traversable cells (obstacles). The UAV estimates the red zone—the area $M_{E}$ that includes all cells with a mean gas concentration $P\left(M_{i}\right)$ exceeding a predetermined threshold $P_{TH}$. The performance evaluation of the red zone estimation method is carried out by comparing $M_{E}$ to the ground-truth red zone $M_{O}$.

### 3.5. Evaluation Metrics

The performance of the sampling path planning approach is evaluated by these suggested metrics: precision, recall, F1, and task duration. The evaluation process requires an initial classification process for each cell in the task zone ($M_{i})$ as True-Positive (TP), False-Positive (FP), and False-Negative (FN).

The TP cells are those that have been classified correctly, as found in $M_{O}\cap M_{E}$. In contrast, the FP cells are found inside the estimated red zone but not inside the ground truth $M_{E}\backslash M_{O}$. Lastly, the FN cells are those found in the ground truth but not inside the estimated area $M_{O}\backslash M_{E}$.

The precision of the presented method is defined here as the ratio of the classified area to the correctly classified area. This is measured as the number of cells that were correctly classified as hazardous $M_{O}\cap M_{E}$ divided by the total amount of the estimated hazardous cells $M_{E}$. This metric assesses the proportion of correct estimations of danger areas TP compared to the total number of positive estimates (TP and FP):

(2) $$\mathrm{precision}=\frac{\mathrm{TP}}{\mathrm{TP}+\mathrm{FP}}=\frac{M_{O}\cap M_{E}}{M_{E}}$$

The recall metric evaluates the ratio of the agent red zone estimation $M_{O}\cap M_{E}\;\left(\mathrm{TP}\right)$ to the total number of red zone cells in the task zone $M_{O}$ (TP and FN):

(3) $$\mathrm{recall}=\frac{\mathrm{TP}}{\mathrm{TP}+\mathrm{FN}}=\frac{M_{O}\cap M_{E}}{M_{O}}$$

In an emergency scenario, the recall is the most important metric representing the ratio between the ground-truth red zone area and the estimated red zone.

Regarding the F1 measure, the metric evaluates the accuracy performance by considering the relative fraction between the union of the ground truth and the estimated red zone $M_{O}\cup M_{E}$ versus the intersection between these areas $M_{O}\cap M_{E}$:

(4) $$\;F1=\frac{\mathrm{TP}}{\mathrm{TP}+\frac{1}{2}\left(\mathrm{FP}+\mathrm{FN}\right)}=\frac{M_{O}\cap M_{E}}{M_{O}\cup M_{E}}$$

Lastly, the duration metric, which examines the feasibility of the task, is defined as Tt—the convergence time of the total area estimation $M_{E}$.

## 4. Path Planning

This section presents the BREEZE path-planning approach, which is described in detail in the following subsections.

### 4.1. Boundary Red Emissions Zone Estimation (BREEZE)

**Figure 3:**
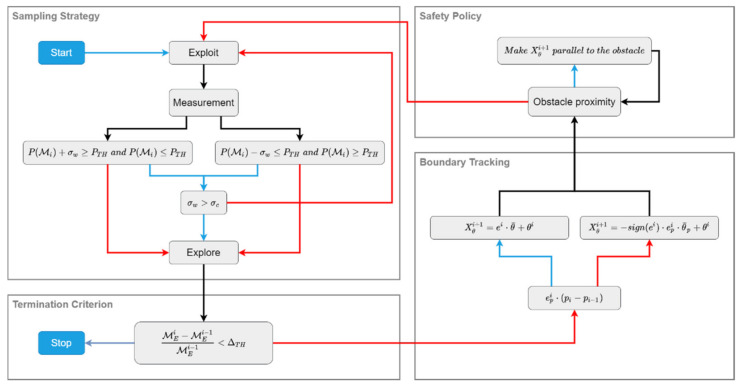
The BREEZE approach. The red and blue arrows in the diagram represent false (red) and true (blue) conditions.

The BREEZE approach is a method for red zone estimation in cluttered environments that finds the boundary of the hazardous area. The diagram in Figure 3 provides a detailed overview of the method that includes four main processes: (1) sampling strategy, (2) boundary tracking, (3) safety policy for obstacle avoidance, and (4) termination criterion.

The sampling strategy process follows two policies that guide the UAV’s action: exploration and exploitation. In the exploration policy, the UAV performs a quick survey of the task zone to reach the proximity of the red zone boundary. In contrast, in the exploitation policy, the UAV continuously measures each cell until a high certainty is achieved regarding the cell’s gas density.

The boundary-tracking process includes a path planner that guides the agent according to the collected measurements. This process aims to produce a path that closely tracks the boundary curve throughout the task.

The safety policy is specifically designed to perform navigation in cluttered environments and to overcome inconsistent gas densities. This process is implemented in sequence with the boundary-tracking process, enabling the UAV to avoid obstacles in its path.

Lastly, the termination criterion process is applied. In this process, the UAV performs area approximation for the red zone and determines whether it is completed before terminating that task.

### 4.2. Sampling Strategy

The BREEZE sampling strategy is a two-policy approach designed to shorten the task duration and to ensure estimation accuracy (see the sampling strategy block in Figure 3).

In the first stage, the agent measures a predetermined number of samples $n_{s}$ and estimates the mean value and standard deviation (std) of the cell in its current location $M_{i}$:

(5) $$\bar{P\left(M_{i}\right)}=\frac{\sum_{i}^{n_{s}}p_{i}}{n_{s}}\;$$

(6) $$\sigma_{f}=\sqrt{\frac{1}{n_{s}}\left(\sum \bar{P\left(M_{i}\right)}-p^{i}\right)}\;$$

These quantities are used to classify the suspected cells, which are defined as those with a gas density interval that includes the threshold ($\;P_{TH}\;\in \left[\bar{P\left(M_{i}\right)}-\sigma_{f},\bar{P\left(M_{i}\right)}+\sigma_{f}\right])$.

In the second stage of the sampling, the cells’ gas density is updated according to:

(7) $$\bar{P\left(M_{i}\right)}=\bar{P\left(M_{i}\right)}+\frac{p^{i}-\bar{P\left(M_{i}\right)}}{N}\;$$

where $p^{i}$ is the current measurement, $\bar{P\left(M_{i}\right)}$ is the cell’s mean gas density value, and N is the number of prior cell measurements. During the classification of the suspected cells, an explore–exploit policy is performed. For unsuspected cells, the exploration policy continues to for the threshold boundary. When a suspected cell is found, the exploitation policy continues to sample the cell until the measured gas density interval equals $\sigma_{c}$.

### 4.3. Boundary Tracking

The BREEZE approach enables the efficient classification of the task zone into safe and hazardous areas by finding the boundary curve of the red zone. To find the red zone boundary curve, the UAV employs an adaptive tracking method. The tracking method performs two policies: Equation (1) closing the gap between the UAV’s current location and the boundary curve, and Equation (2) accurately following the boundary curve.

The gas density error $e^{i}$ is defined as

(8) $$\;e^{i}=p^{i}-P_{TH}\;$$

where $p^{i}$ is the current gas measurement. Finding the boundary curve is based on the proportional policy:

(9) $$\;X_{\theta }^{i+1}=e^{i}\bar{\theta }+\theta^{i}\;$$

where $\bar{\theta }$ is a quantity used to moderate the orientation change, and $X_{\theta }^{i+1}$ is the new orientation of the UAV. This policy directs the UAV from its current location toward the boundary curve.

A second policy is used to follow the boundary curve based on the error between two consecutive measurements. This quantity indicates the deviation from the boundary curve:

(10) $$e_{p}^{i}=p^{i}-p^{i-1}\;$$

**Figure 4:**
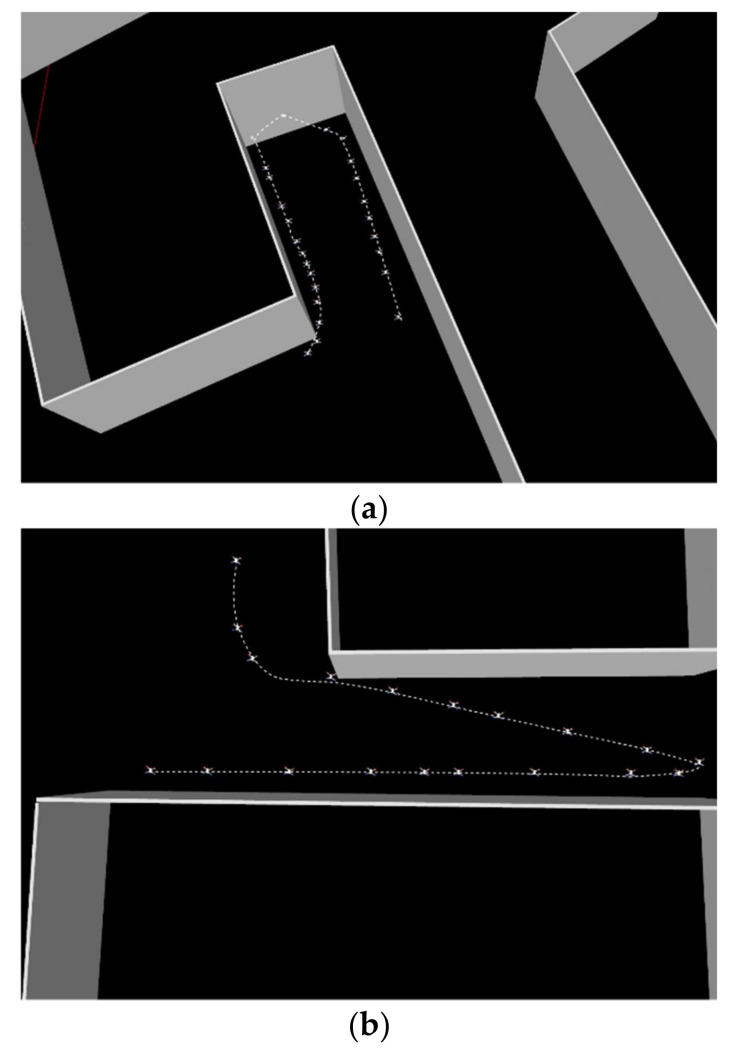
Examples of safety trajectories in proximity to obstacles. The two scenarios presented in subfigures (**a**,**b**) display the ability of the UAV to avoid collisions and safely navigate inside narrow passages.

Accordingly, the boundary tracking policy is calculated as

(11) $$X_{\theta }^{i+1}=-sign\left(e^{i}\right)e_{p}^{i}{\bar{\theta }}_{p}+\theta^{i}\;$$

where ${\bar{\theta }}_{p}$ moderates the orientation changes, and $sign\left(e^{i}\right)$ indicates the UAV’s direction. These policies are activated according to the condition $sign\left(e^{i}\right)\cdot \left(p^{i}-p^{i-1}\right)$, as described in the boundary-tracking block in Figure 4. This capacity enables the smooth tracking of the boundary curve and the continuous moderation of the UAV’s maneuvers throughout the task.

### 4.4. Safety Policy

The BREEZE approach employs a safety policy when the UAV is in close proximity to obstacles. This condition poses two significant risks: (1) collision with obstacles and (2) failure in red zone estimation due to turbulency and inconsistent gas distribution. 

To overcome the risk of collisions, the safety policy uses a binary grid map containing the obstacles’ locations. In each iteration, the policy examines whether the UAV approaches an obstacle cell or not (Figure 4). In cases where the planned motion is inside the obstacle cell, the policy rotates the UAV to be parallel to the obstacle. 

Dealing with inconsistent gas distribution relies on a mechanism applied when the UAV crosses the red zone boundary into the safe zone. This condition is indicated by a change in the value of $sign\left(e^{i}\right)$. When activated, the mechanism guides the UAV to stop following the obstacle edges and to return to performing boundary tracking.

### 4.5. Termination Criterion

The termination criterion process is applied in predetermined periods of time. To determine whether a task is completed or not, the UAV performs area approximation for the red zone $M_{E}$ and calculates the difference between the two consecutive estimations $\Delta =\frac{M_{E}^{i}-M_{E}^{i-1}}{M_{E}^{i-1}}$. When the estimation area change Δ is smaller than a predefined value $\Delta_{TH}$, the red zone estimation is completed, and the task is terminated.

## 5. Red Zone Estimation

The following section describes the simulation setup in detail and presents the results of the simulations using the BREEZE approach. 

### 5.1. Simulation Setup

**Figure 5:**
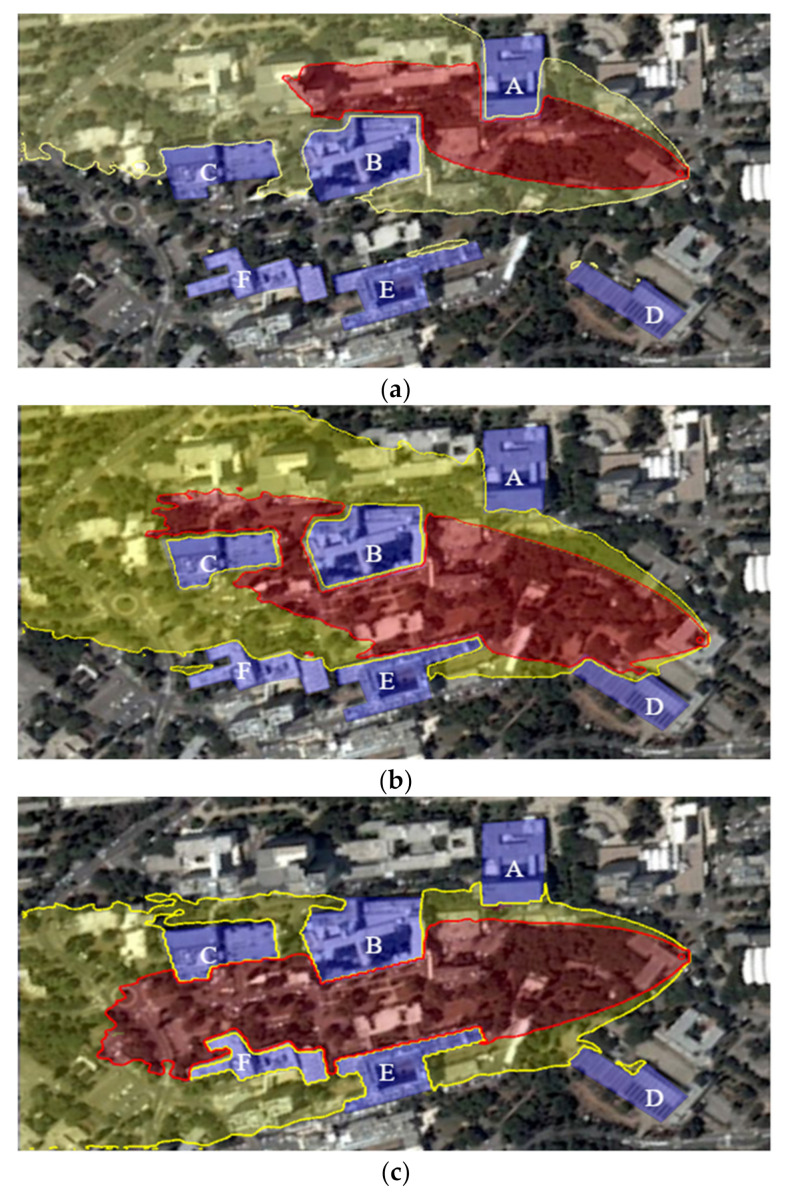
The three tested scenarios: (**a**) Orthogonal Passage, (**b**) Narrow Passage, and (**c**) Corridor. The six buildings inside the task zone are marked with the letters A–F.

The simulation setup includes a realistic environment containing six buildings precisely modeled using Google Earth. Additionally, a gas dispersion map of the environment is generated using the Graz Lagrangian Model. The simulation includes a gas-sensing UAV platform with complete knowledge of its state (location and orientation) as well as of the task zone map. The six-building environment is used to evaluate the performance of the BREEZE approach in varying conditions and obstacle configurations (Figure 5).

Three gas dispersion scenarios were tested: (1) Orthogonal Passage, (2) Narrow Passage, and (3) Corridor. All scenarios were tested with a consistent wind velocity of 5 m/s and varying wind directions.

The Orthogonal Passage scenario (Figure 5a) was tested with a 0° wind direction and two buildings inside the red zone area (A, B). This scenario examines the ability of the BREEZE approach to follow a boundary curve that includes two obstacles that change the plume shape of the gas pollution. 

The Narrow Passage scenario (Figure 5b) was tested with a wind direction of 340° and four buildings inside the red zone area (B–E). In contrast to the Orthogonal Passage scenario, the Narrow Passage scenario examines the ability of BREEZE to recognize the continuity of the pollution while tracking its boundary beyond the obstacles.

Lastly, the Corridor scenario (Figure 5c) was tested with a wind direction of 320° and four buildings inside the red zone (B, C, E, and F). This configuration leads to a long and narrow emission plume between the buildings.

### 5.2. Results

The following section summarizes the results of the simulations according to the three scenarios described above. Each scenario was tested in 10 simulations with different initial conditions, and the results were presented using the mean value $\bar{\mu }$ and the standard deviation $\bar{\sigma }$ of the Evaluation Metrics.

**Figure 6:**
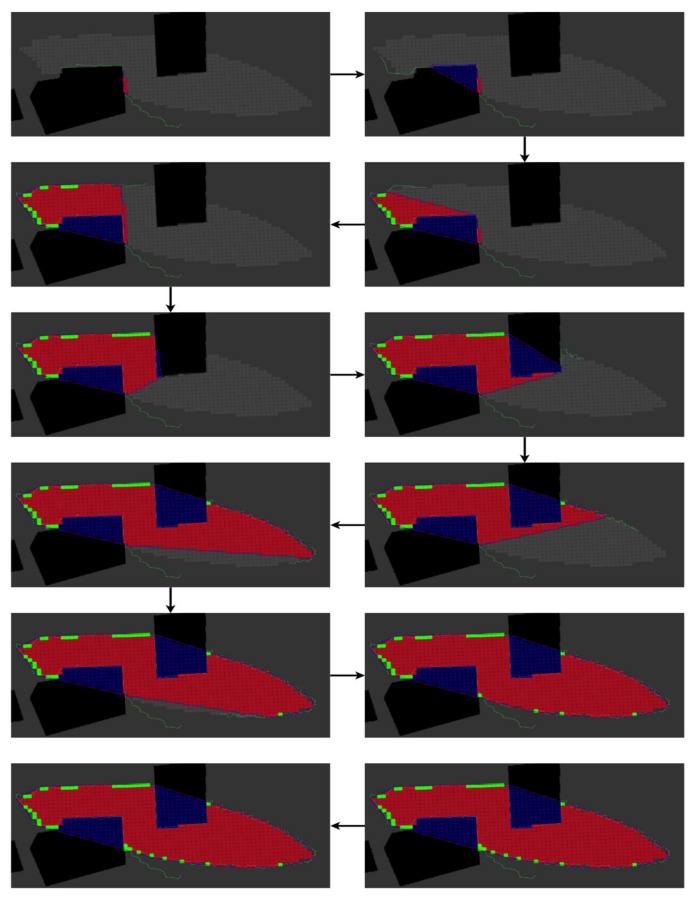
The red zone pollution estimation in the Orthogonal Passage scenario performed using the BREEZE approach with Gaussian noise of 0.5 mg/m^3^. The grey cells are ground truth, the red cells are True-Positive, the green cells are False-Positive, and the blue cells are obstacles.

The results of the Orthogonal Passage scenario are presented in Figure 6 and Table 1. The UAV’s path in this scenario begins outside the red zone, reaches the threshold density, and continues by following the boundary curve and obstacles (the buildings’ walls). 

**Figure 7:**
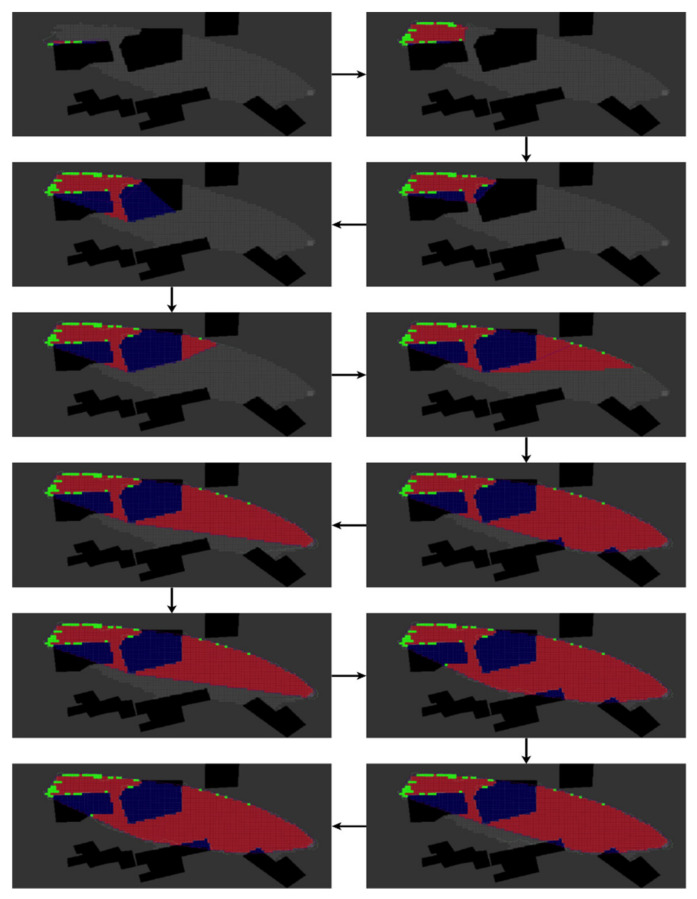
The red zone estimation of the Narrow Passage pollution scenario performed using the BREEZE approach with a Gaussian noise of 0.5 mg/m^3^. The grey cells are ground truth, the red cells are True-Positive, the green cells are False-Positive, and the blue cells are obstacles.

The results of the Narrow Passage scenario are presented in Figure 7 and Table 2. In this scenario, the UAV begins its task in the small patch area, recognizes the narrow passage, and continues to map the primary pollution. This represents the ability of BREEZE to accurately follow the boundary in a confined and non-convex plume shape.

**Figure 8:**
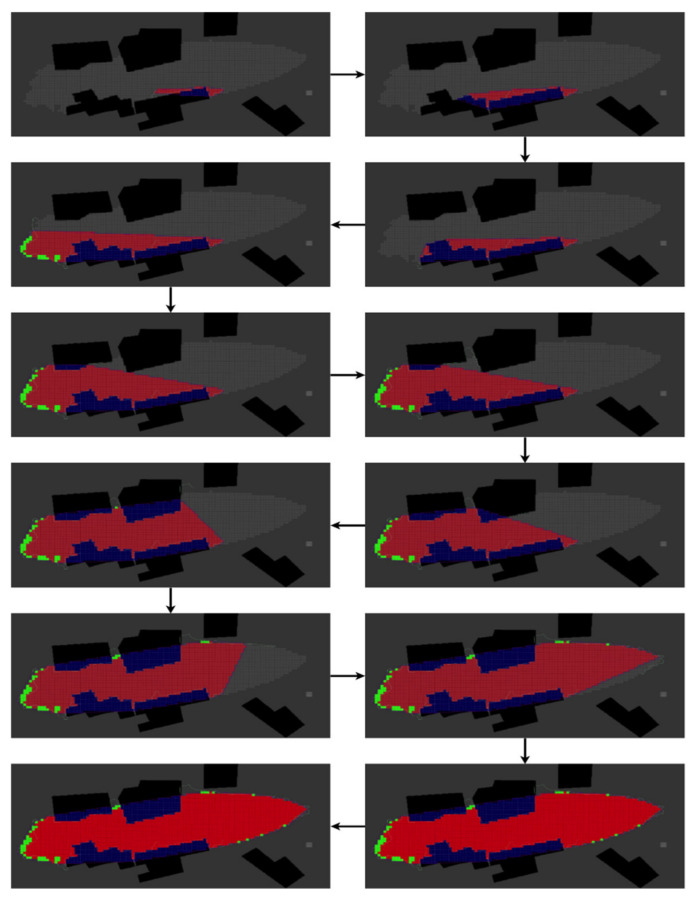
TThis diagram represents the red zone estimation of the Corridor pollution scenario performed using the BREEZE approach with a Gaussian noise of 0.5 mg/m^3^. The grey cells are ground truth, the red cells are True-Positive, the green cells are False-Positive, and the blue cells are obstacles.

Lastly, the results of the Corridor scenario are presented in Figure 8 and Table 3.

Here, the UAV follows the red zone precisely in a cluttered environment that frequently alternates between the safety policy and the boundary-tracking strategy.

The metrics results of the three scenarios present the robustness of the BREEZE approach in achieving accurate red zone estimation compared to the ground truth data (specifically shown by the std and mean values).

### 5.3. Limitation and Conclusions

The BREEZE approach allows the red zone area to be estimated by following its boundary. This significantly shortens the task time while providing accurate estimations. In this context, the simulation results present the robustness of the approach in challenging conditions, varying obstacle configurations, and inconsistent gas dispersion. 

However, a few additional factors need to be considered. While the gas dispersion in the examined scenarios is in a steady state, factors such as changing wind direction could alter the gas dispersion and therefore produce different red zones. This limitation could be overcome by adjusting the criterion to perform sequential red zone estimations and to terminate the process when congruency between two consecutive plumes is achieved.

Furthermore, as shown in the Narrow Passage and Corridor scenarios, the task time increased considerably, in accordance with a rise in noise levels (as seen in Table 1, Table 2 and Table 3). This limitation presents a tradeoff between the task time and confidence threshold (represented by the sampling strategy). Therefore, while reducing the task time is possible, it could potentially reduce the accuracy of the estimation.

## 6. Future Work

This study is part of ongoing research aimed at developing tools and methods for gas sensing and red zone estimation. Future work will expand this research by performing red zone estimation experiments in real-world conditions using a gas-sensing UAV platform guided by the BREEZE approach.

Expanding the research on red zone estimation problems can improve current strategies and provide new problems to be explored. These problems include multi-source gas leaks, additional plume shapes, and multiple red zones in the task zone. Specifically, future research will explore transient conditions that require fast estimation due to frequent changes in the plume shape and will develop 3D estimation strategies aiming to estimate entire gas plume envelopes. 

Red zone estimation can benefit from using multi-agent gas-sensing teams that shorten the task time and increase its performance. Therefore, enhancing the estimation of the red zone requires the development of new cooperation strategies for multiple UAVs.

## Figures and Tables

**Table 1:** The data gathered in the Orthogonal Passage scenario for three different noise levels ($0.5,\;1.0,\;\mathrm{and}\;1.5\;\mathrm{mg}/m^{3}$ ).

$\;\mathrm{Noise}\;\mathrm{mg}/m^{3}$	0.5		1.0		1.5	
Metric	μ	σ	μ	σ	μ	σ
Precision	0.9824	0.0071	0.9677	0.0136	0.9716	0.0189
Recall	0.9533	0.0227	0.9501	0.0204	0.9439	0.0258
$F_{1}$	0.9675	0.0124	0.9586	0.0043	0.9573	0.0139
Time (min)	13.53	0.33	13.65	0.62	14.35	0.71

**Table 2:** The data gathered in the Narrow Passage scenario for three different noise levels (0.5, 1.0, and 1.5 mg/m^3^).

$\;\mathrm{Noise}\;\mathrm{mg}/m^{3}$	0.5		1.0		1.5	
Metric	μ	σ	μ	σ	μ	σ
Precision	0.9248	0.0099	0.9362	0.0179	0.9163	0.0257
Recall	0.9802	0.0047	0.9575	0.0229	0.9669	0.0098
$F_{1}$	0.9517	0.0049	0.9463	0.0030	0.9407	0.0132
Time (min)	18.0	0.21	21.7	0.31	23.2	0.33

**Table 3:** The data gathered in the Corridor scenario for three different noise levels ($0.5,\;1.0,\;\mathrm{and}\;1.5\;\mathrm{mg}/{\;m}^{3}$ ).

$\;\mathrm{Noise}\;\;\mathrm{mg}/m^{3}$	0.5		1.0		1.5	
Metric	μ	σ	μ	σ	μ	σ
Precision	0.965	0.007	0.951	0.024	0.930	0.020
Recall	0.977	0.004	0.981	0.006	0.978	0.013
$F_{1}$	0.971	0.004	0.966	0.010	0.953	0.015
Time (min)	23.971	1.53	29.4	2.215	30.8	3.86
